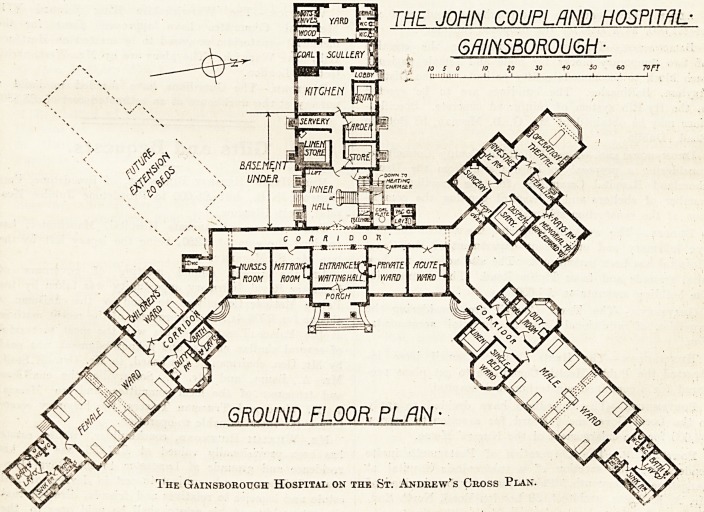# The John Coupland Hospital, Gainsborough

**Published:** 1915-04-10

**Authors:** 


### HOSPITAL ARCHITECTURE AND CONSTRUCTION.

### The John Coupland Hospital, Gainsborough.

This new hospital for Gainsborough and eight adjoining parishes was established in compliance with the provisions of the will of the late Mr. George Coupland and dedicated to the memory of his father.

The site is 3½ acres in extent, and is protected on the east by elevated woodlands, while to the west it has extensive views over wide stretches of pasture lands.

It will be seen from the plan that the building will, when complete, be in the form of a St. Andrew's cross, with an extra arm in the centre.

The centre building is almost entirely administrative, the two wings which project to the north-east and south-east respectively are ward blocks, and the wing projecting north-west is the operation block.

The main entrance is in the centre of the administration block, facing nearly due east. The large entrance hall serves also the purpose of a waiting hall and gives access to the main corridor. To the right of the hall is a private ward and an "acute ward." The precise object of the latter is not clear. For the service of these wards there is a small annex on the other side of the corridor, containing a w.c. and a lavatory. We should have thought that a bed-pan sink would have been of more service here than a lavatory basin, and, in any case, it would have been better for many reasons to have incorporated these wards with one other of the ward wings.

The Gainsborough Hospital on the St. Andrew's Cross Plan.

On the left hand of the entrance is the matron's sitting room and a common sitting room for nurses.

At the back a pair of wide folding doors give access to the main staircase; the precise object of planning this staircase of such generous proportions is not easy to see, or what is gained by making the upper flights in duplicate. On the plan it looks like a very extravagant and quite unnecessary waste of space.

The kitchen offices extend at the back and are of the ordinary type.

The male ward wing contains a large ward for ten beds, a small ward for one bed, nurses' duty room, linen store, storeroom, and coal store. The sanitary offices are at the further end of the ward. These offices comprise a bathroom, two w.c.s, a sink room, and a store for patients' clothes, and the whole block occupies space that would have been put to far better use if it had been occupied by a wide balcony. The sanitary block could with much advantage have been projected from the entrance end of the ward, where it would have been accessible from the small ward, and the bathroom should have been placed in the main building and not in the sanitary annex. It ought not to be necessary to point out in these days that a bathroom does not require disconnecting from the ward in the same way as a w.c. or sink room, and that it is not desirable that its approach should be by way of a draughty passage.

The female ward block is similar to the other, except that it has a ward for five children, and that to make room for this the stores and linen room have been omitted. The same arrangement of sanitary offices obtains here as in the male ward. The operation theatre block contains the theatre, anæsthetic room, sterilising room, surgeons' room, *x*-ray room, and dispensary.

What business the dispensary has in the theatre block it is hard to say; it would have been better to have utilised the space for a wash-up room and so have removed the sinks and lavatories from the theatre, where they ought not to be.

An upper storey over part of the administration block shows a large room called "Committee or Recreation Room," two large and two small bedrooms for nurses and two bedrooms for servants, with two bathrooms and a boxroom. The two large bedrooms are intended to be used as wards in the future. If this ever happens, it is to be hoped that more adequate sanitary provision will be made than is shown on the plan. What provision will be made for nurses when these rooms are turned into wards does not appear.

In the general lay-out of the plan there is much to be commended; with more practical knowledge of the work of hospitals it might have been made a really excellent one. The architect is Mr. William Eyre, of Gainsborough.

## Figures and Tables

**Figure f1:**
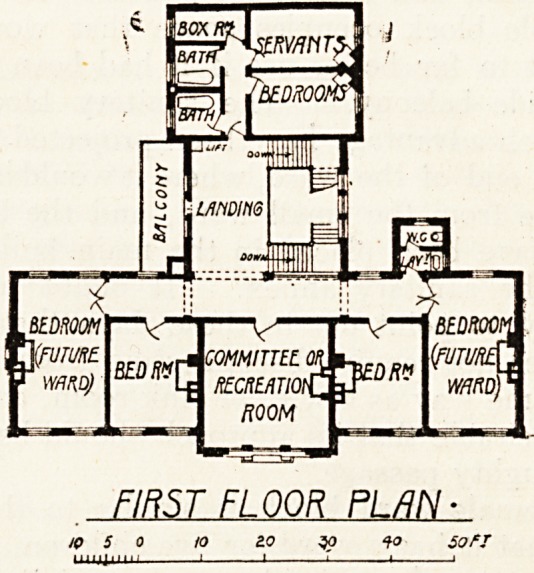


**Figure f2:**